# Exploring suicidal thoughts among prospective university students: a study with applications of machine learning and GIS techniques

**DOI:** 10.1186/s12888-025-07188-2

**Published:** 2025-08-01

**Authors:** Mohammed A. Mamun, Firoj Al-Mamun, Md Emran Hasan, Nitai Roy, Moneerah Mohammad ALmerab, David Gozal, Md. Shakhaoat Hossain

**Affiliations:** 1 Department of Public Health and Informatics, Jahangirnagar University, Dhaka, Bangladesh; 2 Department of Public Health, University of South Asia, Dhaka, Bangladesh; 3 CHINTA Research Bangladesh, Savar, Dhaka, 1342 Bangladesh; 4 School of Computer Science and Engineering, Nanjing University of Science and Technology, Nanjing, 210094 China; 5 Department of Biochemistry and Molecular Biology, Patuakhali Science and Technology University, Patuakhali, Bangladesh; 6 Department of Psychology, College of Education and Human Development, Princess Nourah Bint Abdulrahman University, Riyadh, Saudi Arabia; 7 Office of The Dean and Department of Pediatrics, Joan C. Edwards School of Medicine, Marshall University, 1600 Medical Center Dr, Huntington, WV 25701 USA

**Keywords:** Suicidal behavior, University students, Spatial analysis, Machine learning, CatBoost, Predictive modeling

## Abstract

**Background:**

Prospective university students are regarded as highly vulnerable to psychological issues, including suicide. Despite the complexity of suicidal behaviors, innovative methodologies like Geographic Information System (GIS) mapping and Machine Learning have not been fully explored for predictive modeling and risk assessment. This study aims to investigate the prevalence and risk factors associated with suicidal behavior, offering a thorough understanding of the spatial distribution and predictive factors of suicidality.

**Methods:**

Data from 1,485 participants were collected on socio-demographic characteristics, admission-related variables, health behaviors, and familial factors. Logistic regression analysis identified significant risk factors, while Machine Learning algorithms, including CatBoost and K-Nearest Neighbors, were used for prediction.

**Results:**

The findings revealed a 20.5% prevalence of suicidal thoughts, with disparities across demographics and behaviors. Female participants, rural dwellers, and those from joint families exhibited higher suicidality rates. Repeat test-takers, academically struggling students, and those not coached professionally displayed elevated risks. Moreover, substance use, mental health issues, and family mental health and suicide history increased odds of suicidal behavior. GIS mapping identified regional variations, notably in the Sylhet division and Chittagong Hill Tracts. While, Machine Learning models were used to predict suicidal thoughts, with depression status as the most influential factor. Among all models, CatBoost achieved the best overall performance, with the lowest log loss, highest AUC, and strongest 95% confidence interval. KNN also performed well in accuracy, precision, and F1-score, but showed a slightly higher log loss, making CatBoost the most reliable model for predicting suicidal thoughts.

**Conclusions:**

This study emphasizes the multifaceted nature of suicidal behavior, emphasizing the need for targeted interventions and support services to address mental health challenges and prevent suicide in this vulnerable population.

## Introduction

Every year, approximately 700,000 suicides are reported globally, translating to one death every 40 s [1]. Alarmingly, around 39% of these cases occur in South Asia, with a suicide rate of 17.7 per 100,000 population [2]. In India alone, the suicide rate was recorded at 10.2 per 100,000 in 2018 [3]. Similarly, in Bangladesh, where our present case study is conducted, over 10,000 suicides are reported annually, resulting in a rate of 7.3 per 100,000 [4]. During the COVID-19 pandemic in the country, the prevalence of suicidal ideation was reported to be between 5% and 19%, leading to an 18.5% prevalence of suicidal plans and a high suicide risk for 33.5% of participants, with an expected escalation over time. At least 14,438 suicide cases were reported within one year of the pandemic inception [5].

Suicide is recognized by the World Health Organization as the fourth leading cause of death among individuals aged 15 to 29 [6]. Numerous studies have been conducted to understand suicidal behavior in different populations. For instance, a cross-national study across 12 countries reported that 28.8% of university students had suicidal thoughts, and 7% among them had attempted suicide [7]. Similarly, a systematic review and meta-analysis focusing on young university students found a prevalence of suicidal ideation at 27.1%, with 3.1% reporting suicide attempts [8]. Before the pandemic, Bangladeshi students reported rates of past-year suicidal ideation at 13.4%, with 6.0% reporting lifetime suicide plans and 4.4% attempting suicide [9]. Adolescents in Bangladesh face a particularly high risk, with a suicide rate of 11.3 per 100,000 [4].

Transitioning from high school to university is considered a significant milestone, shaping individuals’ lives with new skills and knowledge [10]. In many countries, including Bangladesh, high school graduates must undergo entrance exams for tertiary education [11]. However, this transition period poses risks to adolescents’ mental health, given the significant educational, social, and emotional changes involved [11, 12]. The competitive nature of these exams, coupled with the extensive preparation required, can significantly impact students’ mental well-being. There are two previous studies that was conducted within university entrance test-taking students, reported high rates of depression, anxiety, and burnout symptoms, closely linked to suicidal tendencies [11, 13]. Before the pandemic, 17.7% of students admitted to contemplating suicide, with 8.0% reporting plans and 2.5% attempting [11], while during the pandemic, prevalence rates were 14.4%, 7.4%, and 7.2%, respectively [13]. However, the post-pandemic scenario remains unexplored, which is the focus of our study.

The Stress-Diathesis Model offers a robust framework for understanding suicidal behavior among university entrance test-takers [14]. This model posits that suicide results from an interaction between environmental stressors and individual vulnerabilities, known as diatheses. In this context, stressors include socio-demographic factors (e.g., gender, residence) as well as academic factors like repeat test-taking, poor academic performance [11, 13]. For instance, female students often face heightened societal and academic pressures, which increases their vulnerability to suicidal ideation [15]. Students from rural areas may experience stress due to limited access to resources, while those in joint families may encounter greater familial obligations and social expectations, compounding their stress [16]. Repeated attempts to pass entrance exams have also been linked to heightened anxiety and a sense of inadequacy [11, 13]. On the other hand, diatheses refer to underlying health and behavioral vulnerabilities, such as substance use, depression, and anxiety, that may predispose individual to adverse responses under stress [17]. Guided by this framework, the present study examines how the interaction between these stressors and vulnerabilities contributes to suicidal thoughts among Bangladeshi university entrance examinees [18].

Recent advances in suicide prediction and prevention point to the potential contributions of artificial intelligence and Machine Learning (ML) techniques, since these have proven effective in assessing risk factors for suicidal behaviors. A systematic review analyzing 156 studies from 2019 to 2023, highlights AI methods such as ML, deep learning, and natural language processing for improving risk assessment accuracy through multimodal data [19]. In addition, Kusuma et al. [20] conducted a systematic review and meta-analysis on ML’s effectiveness in predicting suicidal ideation, attempts, and deaths, yielding a pooled AUC of 0.86, high specificity (0.87), and moderate sensitivity (0.66) across 54 models from 35 studies. Another systematic review investigated ML applications specifically for suicide risk prediction within psychiatric populations, addressing mixed-population limitations and identifying key predictors like previous attempts, disorder severity, and medication history [21]. Models such as random forest and support vector machines achieved over 70% accuracy, but emphasized the need for neurobiological and imaging data and external validation to strengthen ML’s clinical impact in suicide prevention.

Machine Learning studies among student and youth populations worldwide have identified consistent predictors of suicidality, shaped by cultural and demographic factors. In China, a study of 4,882 medical students found anxiety, depression, suicidal ideation, suicide plans, and family relationships emerged as significant predictors of suicide attempts, with the Random Forest model achieving 90.1% accuracy, 73.51% sensitivity, and 91.68% specificity [22]. In the Middle East and North Africa, traumatic experiences, PTSD symptoms, and low social support were major risk factors identified through three-fold cross-validation [23], while Korean adolescent data highlighted mental health, academic stress, and social factors as significant predictors of suicidal ideation [24]. A French longitudinal study reported AUCs of 0.828–0.829 across logistic regression, lasso, ridge, and random forest models [25]. Huang et al. [26] analyzed data from 10,243 Chinese adolescents and found emotional neglect and poor social support to be influential,, while another study noted aggressive behavior and bullying as gender-specific risks among college students [27]. In younger cohorts, a study within a total of 4,691 Chinese preadolescents identified internalizing/externalizing problems, neuroticism, and school well-being as predictors of suicidal ideation [28]. Among 5,066 French college students, 17% reported suicidal thoughts, with past ideation, trait anxiety, depression, and low self-esteem as the top predictors [29]. A large-scale U.S. study involving 179,000 high school students further emphasized academic performance and peer relationships as critical factors [30], reinforcing the role of ML in early identification and intervention across academic settings.

In Bangladesh, where this study is situated, rising youth suicide rates are a growing public health concern, driven by a combination of social, economic, and academic pressures. Students preparing for university entrance exams experience intense psychological burdens due to the competitive nature of admissions, limited institutional mental health support, and societal expectations of success. While, a few existing studies have explored suicidal behaviors in this group using conventional statistical approaches., there remains a significant methodological gap, no study to date has employed machine learning techniques or geographic information systems to examine predictive risk factors or spatial patterns of suicidality in Bangladeshi youth. Previous studies have identified factors such as gender, residence, academic performance, satisfaction with mock tests, substance use, COVID-19-related factors, and mental health issues as relevant correlates of suicidal thoughts [11, 13], yet other important dimensions, such as the mental health history of students and their families, remain largely overlooked [31]. Furthermore, studies to date have treated suicidality as a static condition, whereas ML and GIS enable dynamic and high-resolution modeling that can reveal nonlinear patterns and geographic clustering. Despite global advancements in these technologies for suicide prevention, they remain underutilized in Bangladesh [19–21]. Using the Stress-Diathesis Model as a conceptual framework, this study address these gaps by applying ML algorithms and GIS-based mapping to a large and diverse sample of university entrance examinees. In doing so, it contributes original insights into the complex psychosocial and spatial determinants of youth suicidality and provides a data-driven foundation for early warning systems and geographically targeted prevention strategies in the Bangladeshi context.

## Methods

### Study participants and procedure

This cross-sectional study was conducted among university students appearing for entrance tests at Jahangirnagar University, situated in Dhaka, Bangladesh. The entrance test took place over a period spanning from 22 to 29 February 2024, and data collection occurred within this timeframe. The study targeted students who were residing in university dormitories during the entrance test period, with specific rooms designated for their accommodation. Employing a convenience sampling approach, all eligible participants present in the dormitories during the survey period were approached and invited to participate.

To ensure efficient data collection, a team of 16 trained personnel, including eight data collectors and eight researchers, worked simultaneously across multiple dormitories. For the data collection in the female dormitories, female team members (consisting of three researchers and five additional data collectors), were responsible for collecting data from female participants. The participants’ availability and the structured setting of the dormitories facilitated access to the students, allowing for a high participation rate within the short seven-day period. Approximately 1600 participants were approached for this study, and informed consent was obtained after providing comprehensive information regarding the study’s purpose and the nature of participation. Initially, a total of 1509 responses were obtained (≈ 98.4% response rate), but after excluding incomplete questionnaires, data from 1485 participants were subjected to analysis.

### Measures

#### Sociodemographic factors

A number of sociodemographic information was collected through the survey, including gender, residence location (urban or rural), monthly family income, and religious affiliation. To gauge socioeconomic status, participants were categorized into three groups based on their monthly family income: lower class (earning less than 15,000 Bangladeshi Taka [BDT]), middle class (earning between 15,000 and 30,000 BDT), and upper class (earning more than 30,000 BDT).

#### Health and behavioral variables

Initially, participants were queried about their engagement with smoking, drug, and alcohol use. Following this, their history of mental health challenges and physical health was evaluated. Besides, participants were asked about any instances of mental health struggles, suicide attempts, or occurrences of suicide within their family circle. All questions were designed to elicit binary responses, where participants indicated either ‘yes’ or ‘no’ to each inquiry.

#### Admission-related variables

Information about the students’ admission journey was collected, encompassing details such as their test-taking status (first-time or repeat test-takers), educational background, Grade Point Average (GPA), involvement of professional guidance or coaching centers in their preparation, performance in mock tests, monthly expenditure related to test preparation, and preference regarding the type of university for admission.

#### Patient health questionnaire

Depressive symptoms were evaluated utilizing the Bangla Patient Health Questionnaire (PHQ-9), comprising nine items that participants responded to based on their experiences over the past two weeks [32, 33]. Scores ranged from 0 to 27, with a higher score indicative of greater severity of depressive symptoms. A cutoff score of 10 or higher was employed to identify individuals experiencing significant depression [32]. In the present study, Cronbach’s alpha was 0.76.

#### Generalized anxiety disorder

Anxiety symptoms were evaluated utilizing the Generalized Anxiety Disorder (GAD-7), which comprised seven items respondents rated based on their experiences over the past two weeks [34, 35]. Scores ranged from 0 to 21, with higher scores indicating more severe anxiety symptoms. A cutoff score of 10 or higher was utilized to identify individuals experiencing significant anxiety [34]. In the present study, Cronbach’s alpha was 0.76.

#### Suicidal thoughts

In the survey, test-taking students were asked about any thoughts of suicide they might have had with a detailed single item with example, serving as an assessment of suicidal thoughts. This questioning approach was derived from a previous study conducted among university entrance test-taking students, which laid the groundwork for evaluating suicidal behaviors [11, 36]. The response was recorded in a binary response (Yes/No).

### Ethical consideration

The authors assert that all procedures contributing to this work comply with the ethical standards in accordance with the Declaration of Helsinki of 1975, as revised in 2008. All procedures involving human subjects were approved by institutional review board at Patuakhali Science and Technology University [Ref: PSTU/IEC/2023/85(2)], which is applicable to study conducted over the country. Prior to participating in the study, all participants were provided with detailed information regarding the study’s objectives, procedures, and potential risks and benefits. The study was designed with careful attention to minimizing potential psychological stress. Validated mental health assessment tools were employed to assess suicidal thoughts, and participants were provided with information about available mental health resources. They also had the option to opt out if they felt uncomfortable. However, informed written consent was obtained from each participant prior to survey administration, underscoring their voluntary participation and right to withdraw from the study at any stage.

### Statistical analysis

For this study, data collection utilized Google Forms, with subsequent formatting for an SPSS file to facilitate final analysis. Descriptive and inferential statistics were employed to analyze the data. Descriptive statistics utilized frequency and percentage calculations, while the chi-square test and binary logistic regression were applied to explore associations between the studied variables and suicidal ideation. Furthermore, ArcGIS 10.8 software was utilized for spatial analysis of suicidal ideation. Initially, total participant data was geographically presented by district. Subsequently, two post-hoc analyses were conducted and illustrated through maps based on gender and student status. The maps were generated using data obtained from government mapping sites, which are freely accessible for use. However, a significance level of *p* < 0.05 was set for all tests, and a standard 95% confidence interval was employed throughout the study.

### Machine learning analysis

In this study, suicidal thoughts among prospective university students were analyzed and predicted using a machine learning techniques approach that was created with methodological rigour, reproducibility, and transparency in mind. The complete procedure was carried out in a methodical, chronological order. To deal with inconsistent entries, outliers, and missing values, data cleaning was done first. Participants were eliminated if their answers were deemed invalid or incomplete. To guarantee consistency among characteristics, continuous variables were standardized. The Synthetic Minority Oversampling Technique (SMOTE) was then used just on the training set to rectify the class imbalance between students who had suicidal thoughts and those who did not [37, 38]. To prevent data leakage and guarantee objective model evaluation, this phase was meticulously limited to prevent synthetic data from polluting the test set. 5-fold cross-validation was used during model training to guarantee an accurate and robust performance estimate. In the initial fold, the class distribution of the training set prior to SMOTE consisted of 949 samples for Target 0 (No Suicidal Thought) and 239 samples for Target 1 (Yes Suicidal Thought). Following the application of SMOTE, both classes were equilibrated with 949 samples each.

Several machine learning models, including K-Nearest Neighbors (KNN), Random Forest (RF), eXtreme Gradient Boosting (XGBoost), Decision Tree (DT), and Categorical Boosting (CatBoost), were then built and trained. 20% of the dataset was set aside for testing, and 80% for training. Models were assessed on the independent test set following training and optimization. Accuracy, Precision, F1-score, Log-Loss, AUC, 95% CI and Area Under the Receiver Operating Characteristic Curve (AUC-ROC) were among the measures used to evaluate the performance. In this analysis also used a bootstrapping method to compare the AUC values between different models. These measures offered a thorough assessment of each model’s capacity for prediction.

The best-performing model was subjected to Shapley Additive exPlanations (SHAP) for model interpretability in order to assess feature relevance and comprehend the ways in which each predictor affected the model’s judgment of suicidal thoughts. Python on Google Colab was used for all calculations and model implementations, making use of its cloud-based processing capabilities for effective processing. Additionally, the learning curves for the machine learning models are depicted. These curves illustrate the learning behaviors of the models and possible overfitting or underfitting patterns by showing the relationship between the number of training examples and the corresponding training and testing accuracies.

Local Interpretable Model-Agnostic Explanations (LIME) was employed to elucidate the predictions of the XGBoost model. LIME elucidates the significance of characteristics for particular test instances, enhancing the transparency and comprehensibility of the model’s predictions. Recursive Feature Elimination (RFE) method was utilized to identify the most critical features by systematically eliminating the least significant ones. It enhances model performance by mitigating overfitting and emphasizing the most essential features. Similarly, Recursive Feature Elimination with Cross-Validation (RFE-CV) was employed to enhance feature selection by guaranteeing that the model is trained on a set of features that are resilient and exhibit strong performance on unseen data, thereby mitigating the danger of overfitting.

The model’s final evaluation included performance indicators including accuracy, precision, F1-score and AUC on the test set. These measures offer a thorough comprehension of the model’s prediction capability. Additionally, confusion matrix of best model was created for the optimal model to evaluate its classification accuracy, displaying true positives, true negatives, false positives, and false negatives.

## Results

### Description of the study participants

Table 1 presents the characteristics of the study participants and their distributions based on suicidal behavior. A total of 1,485 university test-taking students participated, with 58.4% being male and 41.6% female. The majority were from rural areas (62.1%), identified as Muslim (74.8%), belonged to nuclear families (72.9%), and had a monthly family income over 30,000 Bangladeshi Taka (53.1%). In terms of admission-related variables, 53.8% were fresher test-takers, 65.4% had a high Secondary School Certificate GPA of 5, 72.1% were coached by professional coaching centers, and 71.1% aimed for admission to university departments.

**Table 1:** Characteristics of the participants and their distributions by suicidal thoughts

Variables	Total Sample	Suicidal thoughts			
	*n*; (%)	No; *n* (%)	Yes; *n* (%)	χ^2^ test value	*p*-value
Socio-demographic variables					
Gender					
Male	867; 58.4%	709; 81.8%	158; 18.2%	16.909	< 0.001
Female	618; 41.6%	450; 72.8%	168; 27.2%		
Permanent residence					
Rural	922; 62.1%	743; 80.6%	179; 19.4%	8.650	0.003
Urban	555; 37.4	411; 74.1%	144; 25.9%		
Religion					
Muslim	1111; 74.8%	883; 79.5%	228; 20.5%	5.237	0.022
Others	366; 24.6%	270; 73.8%	96; 26.2%		
Family type					
Nuclear	1083; 72.9%	883; 81.5%	200; 18.5%	32.408	< 0.001
Joint	363; 24.4%	244; 67.2%	119; 32.8%		
Monthly family income (BDT)					
< 15,001	194; 13.1%	155; 79.9%	39; 20.1%	10.470	0.005
15,001–30,000	381; 25.7%	319; 83.7%	62; 16.3%		
> 30,000	789; 53.1%	596; 75.5%	193; 24.5%		
Admission-related variables					
Appearance in the admission test					
Fresher test-takers	799; 53.8%	659; 82.5%	140; 17.5%	19.820	< 0.001
Repeat test-takers	686; 46.2%	500; 72.9%	186; 27.1%		
Secondary School Certificate (SSC) grade point average					
Poor (< 4.51)	250; 16.8%	147; 58.8%	103; 41.2%	68.932	< 0.001
Moderate (4.51–4.99)	236; 15.9%	194; 82.2%	42; 17.8%		
High (5)	971; 65.4%	802; 82.6%	169; 17.4%		
Higher Secondary Certificate (HSC) grade point average					
Poor (< 4.51)	236; 15.9%	160; 67.8%	76; 32.2%	22.847	< 0.001
Moderate (4.51–4.99)	315; 21.2%	242; 76.8%	73; 23.2		
High (5)	907; 61.1%	743; 81.9%	164; 18.1%		
Coached by professional coaching centers					
Yes	1070; 72.1%	868; 81.1%	202; 18.9%	21.288	< 0.001
No	399; 26.9%	279; 69.9%	120; 30.1%		
Desired institute/department for admission					
Varsity	1056; 71.1%	856; 81.1%	200; 18.9%	52.208	< 0.001
Medical	261; 17.6%	198; 75.9%	63; 24.1%		
Engineering	118; 7.9%	83; 70.3%	35; 29.7%		
Agriculture	43; 2.9%	16; 37.2%	27; 62.8%		
Satisfied with previous mock tests					
No	756; 50.9%	577; 76.3%	179; 23.7%	1.702	0.192
Yes	585; 39.4%	464; 79.3%	121; 20.7%		
Average monthly expenditure (BDT)					
> 5,000	568; 38.2%	485; 85.4%	83; 14.6%	125.667	< 0.001
5001-10,000	550; 37.0%	459; 83.5%	91; 16.5%		
> 10,000	222; 14.9%	113; 50.9%	109; 49.1%		
Educational background					
Science	955; 64.3%	766; 80.2%	189; 19.8%	10.824	0.004
Arts	309; 20.8%	239; 77.3%	65; 30.0%		
Commerce	217; 14.6%	152; 70.0%	70; 22.7%		
Health and behavioral variables					
Cigarette smoking status					
No	1176; 79.2%	960; 81.6%	216; 18.4%	42.406	< 0.001
Yes	309; 20.8%	199; 64.4%	110; 35.6%		
Drug usage status					
No	1335; 89.9%	1082; 81.0%	253; 19.0%	69.496	< 0.001
Yes	150; 10.1%	77; 51.3%	73; 48.7%		
Alcohol usage status					
No	1338; 90.1%	1079; 80.6%	259; 19.4%	53.149	< 0.001
Yes	147; 9.9%	80; 54.4%	67; 45.6%		
History of mental health problem					
No	1171; 78.9%	951; 81.2%	220; 18.8%	31.818	< 0.001
Yes	314; 21.1%	207; 66.3%	105; 33.7%		
History of physical health problem					
No	1167; 78.6%	940; 80.5%	227; 19.5%	21.022	< 0.001
Yes	314; 21.0%	215; 68.5%	99; 31.5%		
History of mental health problem in family					
No	1300; 87.5%	1051; 80.8%	249; 19.2%	46.003	< 0.001
Yes	175; 11.8%	102; 58.3%	73; 41.7%		
History of suicide attempt in family					
No	1304; 87.8%	1053; 80.8%	251; 19.2%	47.086	< 0.001
Yes	179; 12.1%	104; 58.1%	75; 41.9%		
History of suicide in family					
No	1326; 89.3%	1068; 80.5%	258; 19.5%	44.962	< 0.001
Yes	153; 10.3%	87; 56.9%	66; 43.1%		
Depression status					
No	715; 48.1%	652; 91.2%	63; 8.8%	138.994	< 0.001
Yes	770; 51.9%	507; 65.8%	263; 34.2%		
Anxiety status					
No	992; 66.8%	855; 86.2%	137; 13.8%	115.623	< 0.001
Yes	493; 33.2%	304; 61.4%	189; 38.3%		

*BDT* Bangladeshi Taka

### Suicidal thoughts across districts

**Fig. 1:**
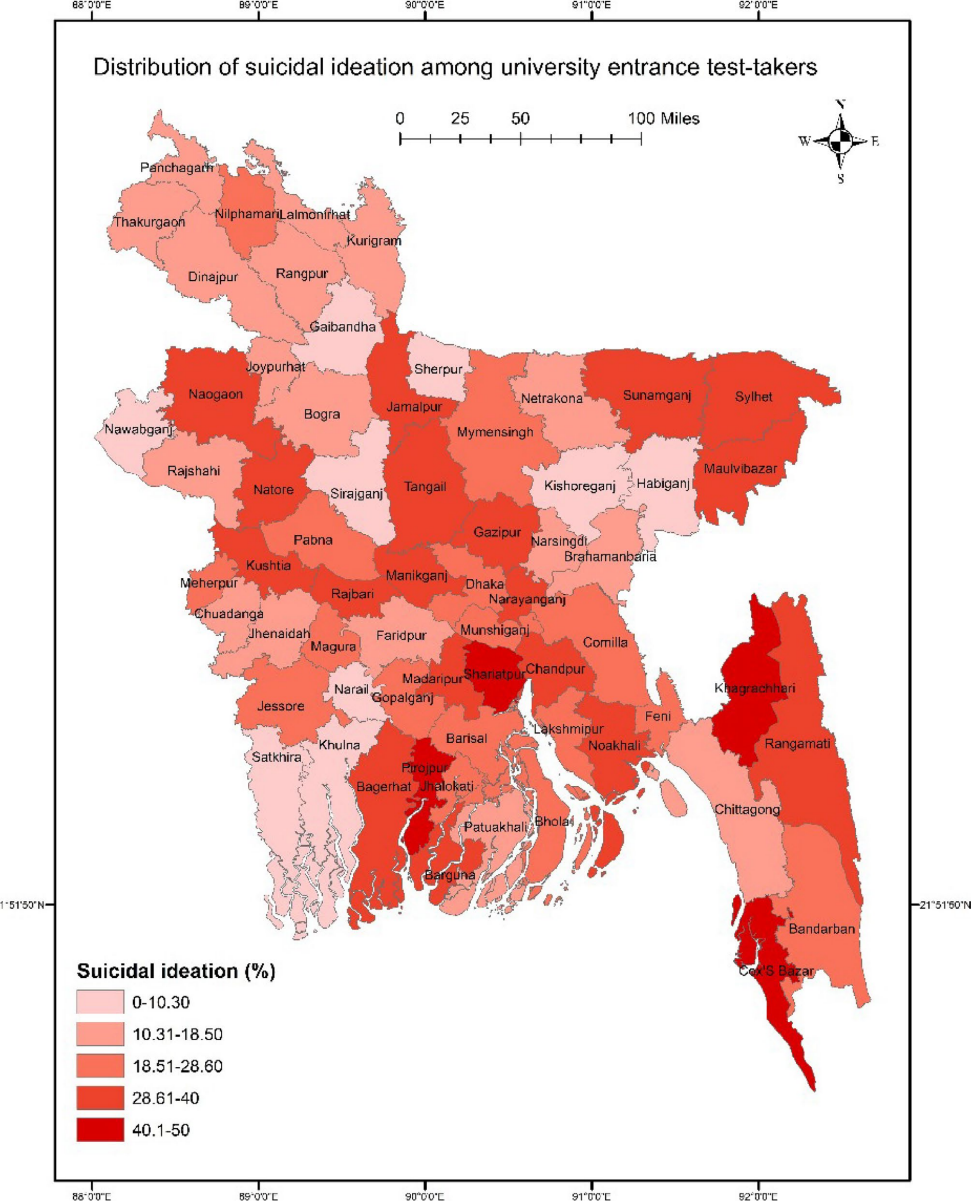
GIS-based distribution of suicidal thoughts among students taking entrance test

The study found that 20.5% of the participants reported experiencing suicidal thoughts in the past year. Spatial analysis revealed a nationwide distribution of suicidal thoughts, with significant variations across districts (χ^2^ = 88.842, *p* = 0.018). Particularly high prevalence rates of suicidality were observed in certain districts, notably those within the Sylhet division such as Sunamganj, Sylhet, and Maulovibazar. Besides, elevated rates were identified in Naogaon, Natore, Shariatpur, Pirojpur, Cox’s Bazar, and districts within the Chittagong Hill Tracts, including Khagrachori and Rangamati (Fig. 1).

### Associations between the variables and suicidal thoughts

Suicidal thoughts were significantly higher among females compared to males (27.2% vs. 18.2%, *p* < 0.001), students from urban areas (25.9% vs. 19.4%, *p* = 0.003) and nuclear families (32.8% vs. 18.5%, *p* < 0.001). Besides, suicidality was reported by 24.5% of the participants from high-income families, 20.1% from low-income families, and 16.3% from middle-income families (*p* = 0.005).

Regarding admission-related variables, repeat test-takers demonstrated a significantly higher prevalence of suicidal ideation compared to fresher test-takers (27.1% vs. 17.5%, *p* < 0.001). In addition, participants with poor Secondary School Certificate (SSC) and Higher Secondary Certificate (HSC) Grade Point Averages (GPAs) displayed higher rates of suicidal thoughts. Moreover, those who were not coached by professional coaching centers reported a higher prevalence of suicidality compared to those who were (30.1% vs. 18.9%, *p* < 0.001). Notably, the desired institute for admission exhibited a significant relationship with suicidal thoughts (*p* < 0.001), with agricultural department aspirants displaying the highest prevalence (62.8%), whereas those aiming for varsity had the lowest rates (18.9%).

In terms of health and behavioral variables, participants who smoked cigarettes (35.6% vs. 18.4%, *p* < 0.001), used drugs (48.7% vs. 19.0%, *p* < 0.001), and consumed alcohol (45.6% vs. 19.4%, *p* < 0.001) exhibited significantly higher rates of suicidal thoughts. Moreover, participants with a history of mental health problems displayed a higher prevalence of suicidality compared to those without such a history (33.7% vs. 18.8%, *p* < 0.001); and similar observation was found for a history of physical health problems (31.5% vs. 19.5%, *p* < 0.001). Notably, family history of mental health problems (41.7% vs. 19.2%, *p* < 0.001), suicide attempts (41.9% vs. 19.2%, *p* < 0.001), and suicide (43.1% vs. 19.5%, *p* < 0.001) also exhibited significantly higher rates of suicidal thoughts. Finally, those students with depression had a prevalence of 34.2% suicidality compared to 8.8% normal ones, which was as 38.3% vs. 13.8% for anxiety status (Table 1).

### Factors associated with suicidal thoughts

The binary logistic regression analysis unveiled numerous significant risk factors linked to suicidal thoughts, as depicted by unadjusted model in Table 2. Notably, females exhibited 1.675 times higher odds of suicidal ideation than males (*p* < 0.001). Conversely, participants hailing from rural areas demonstrated 0.688 times lower odds of suicidal thoughts compared to their urban counterparts (*p* = 0.003). Similarly, individuals identifying with religions other than Islam displayed 1.377 times higher odds of suicidal thoughts (*p* = 0.022), while those from joint families faced 2.153 times higher odds compared to those from nuclear families (*p* < 0.001).

**Table 2:** Logistic regression analysis concerning risk factors associated with suicidal thoughts

Variables	Unadjusted model			Adjusted model		
	OR	95% CI	*p*-value	OR	95% CI	*p*-value
Socio-demographic variables						
Gender						
Male	Reference		<0.001	Reference		0.210
Female	1.675	1.308-2.145		1.276	0.871-1.869	
Permanent residence						
Urban	Reference		0.003	Reference		0.403
Rural	0.688	0.535-.883		1.182	0.799-1.748	
Religion						
Muslim	Reference		0.022	Reference		0.848
Others	1.377	1.046-1.812		1.041	0.687-1.578	
Family type						
Nuclear	Reference		<0.001	Reference		0.052
Joint	2.153	1.648-2.814		1.489	0.997-2.223	
Monthly family income (BDT)						
<15000	Reference		0.006	Reference		0.391
15000-30000	0.772	0.495-1.204		1.338	0.716-2.498	
>30000	1.287	0.874-1.895		0.986	0.541-1.799	
Admission-related variables						
Appearance in the admission test						
Fresher test-takers	Reference		<0.001	Reference		0.009
Repeat test-takers	1.751	1.366-2.244		1.661	1.133-2.436	
Secondary School Certificate (SSC) grade point average						
Poor (<4.5)	Reference		<0.001	Reference		0.008
Moderate (4.51-4.99)	0.309	0.203-0.469		0.530	0.291-0.964	
High (5)	0.301	0.222-0.407		0.451	0.272-0.750	
Higher Secondary Certificate (HSC) grade point average						
Poor (<4.5)	Reference		<0.001	Reference		0.251
Moderate	0.635	0.435-0.927		0.653	0.365-1.168	
High (5)	0.465	0.337-0.641		0.653	0.381-1.118	
Coached by professional coaching centers						
Coaching	Reference		<0.001	Reference		0.027
Self	1.848	1.421-2.404		1.623	1.056-2.494	
Desired institute/department for admission						
Medical	Reference		<0.001	Reference		0.013
Varsity	0.734	0.532-1.014		0.836	0.490=1.427	
Engineering	1.325	0.815-2.155		1.106	0.539-2.272	
Agriculture	5.304	2.686-10.471		3.881	1.440-10.461	
Satisfied with previous mock tests						
No	Reference		0.192	Reference		0.806
Yes	0.841	0.647-1.091		0.952	0.642-1.411	
Average monthly expenditure (BDT)						
<5,000	Reference		<0.001	Reference		<0.001
5001-10,000	1.158	0.838-1.601		1.582	1.036-2.416	
>10,000	5.637	3.967-8.009		5.194	3.045-8.857	
Educational background						
Science	Reference		0.005	Reference		0.043
Arts	1.187	.870-1.619		1.994	1.159-3.430	
Commerce	1.733	1.244-2.414		1.395	0.842-2.309	
Health and behavioral variables						
Cigarette smoking status						
No	Reference		<0.001	Reference		0.231
Yes	2.457	1.865-3.237		1.357	.824-2.237	
Drug usage status						
No	Reference		<0.001	Reference		.004
Yes	4.055	2.862-5.743		2.712	1.386-5.308	
Alcohol usage status						
No	Reference		<0.001	Reference		0.282
Yes	3.489	2.454-4.960		1.436	0.743-2.774	
History of mental health problems						
No	Reference		<0.001	Reference		0.721
Yes	2.193	1.662-2.892		0.915	0.560-1.493	
History of physical health problems						
No	Reference		<0.001	Reference		0.043
Yes	1.907	1.443-2.520		1.665	1.017-2.728	
History of mental health problems in family						
No	Reference		<0.001	Reference		0.301
Yes	3.021	2.170-4.205		1.350	0.764-2.385	
History of suicide attempt in family						
No	Reference		<0.001	Reference		0.207
Yes	3.025	2.181-4.197		.693	0.392-1.225	
History of suicide in family						
No	Reference		<0.001	Reference		0.443
Yes	3.140	2.218-4.446		1.253	0.704-2.229	
Depression status						
No	Reference		<0.001			<0.001
Yes	5.369	3.983-7.235		4.212	2.622-6.767	
Anxiety status						
No	Reference		<0.001	Reference		0.005
Yes	3.880	3.004-5.012		1.772	1.187-2.646	

*BDT* Bangladeshi Taka

Regarding admission-related variables, repeat test-takers exhibited 1.751 times higher odds of suicidal ideation compared to fresher test-takers (*p* < 0.001). Furthermore, participants receiving coaching from professional centers had 1.848 times higher odds of suicidal thoughts than those self-preparing (*p* < 0.001). Notably, participants aspiring for admission to agriculture departments faced significantly higher odds of suicidal thoughts (OR = 5.304, *p* < 0.001) compared to those desiring medical admission. Financial aspects also played a role, with participants spending over 10,000 BDT having 5.637 times higher odds of suicidal thoughts (*p* < 0.001) compared to those spending less.

In terms of behavioral factors, smokers demonstrated 2.457 times higher odds of suicidal thoughts compared to non-smokers (*p* < 0.001). Similarly, the odds were 4.055 times and 3.489 times higher for drug users and alcohol users, respectively. Family history emerged as a significant predictor, with participants having a history of mental health problems, suicide attempts, or suicide in the family showing notably higher odds of suicidal thoughts (*p* < 0.001). Finally, participants with depression had a depression risk of 5.369, which was 3.880 times for anxiety (Table 2).

### Model interpretability using SHAP values

**Fig. 2:**
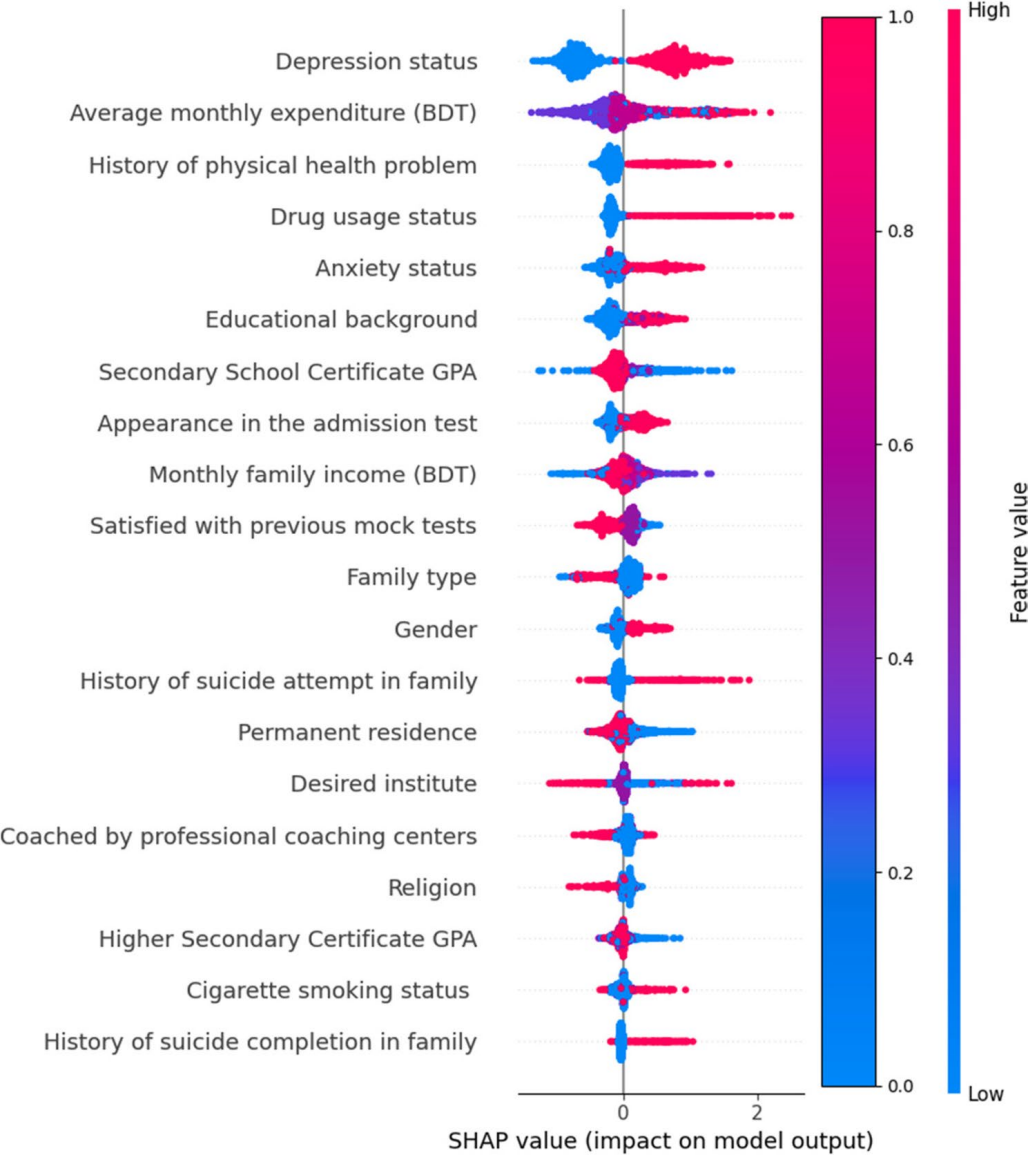
Features impact on the model by SHAP value

XGBoost’s SHapley Additive exPlanations (SHAP) values are used to assess the impact of features, and Cramer’s V criterion is used to analyze feature correlations to choose which features to include in the ML model. Since each attribute had a strong impact on the model’s output and little correlation with one another, all of them were eventually incorporated into the model. Remarkably, it turned out that depression status was the most significant factor, whereas history of suicide in the family had the least effect (Fig. 2). In addition, some characteristics showed comparatively higher influence on the model’s output such as average monthly expenditure (BDT), history of physical health problems, drug usage status, anxiety status, educational background, secondary school certificate GPA, appearance in the admission test, monthly family income (BDT) and satisfied with previous mock tests. The PHQ-9’s great feature importance in the SHAP analysis is indicative of its function in evaluating the severity of depression (including suicidal thoughts). The PHQ-9’s items pertaining to suicidal thoughts are inherently significant in the model’s predictions, as they are a crucial predictor of suicide risk. While the position on the X-axis represents the SHAP value, or the influence on the prediction probability, the color gradient shows the feature values (high in red, low in blue). This study highlights the critical elements linked to the likelihood of suicidal thoughts and offers clear insights into the model’s decision-making process.

### Feature selection using recursive feature elimination (RFE)

**Fig. 3:**
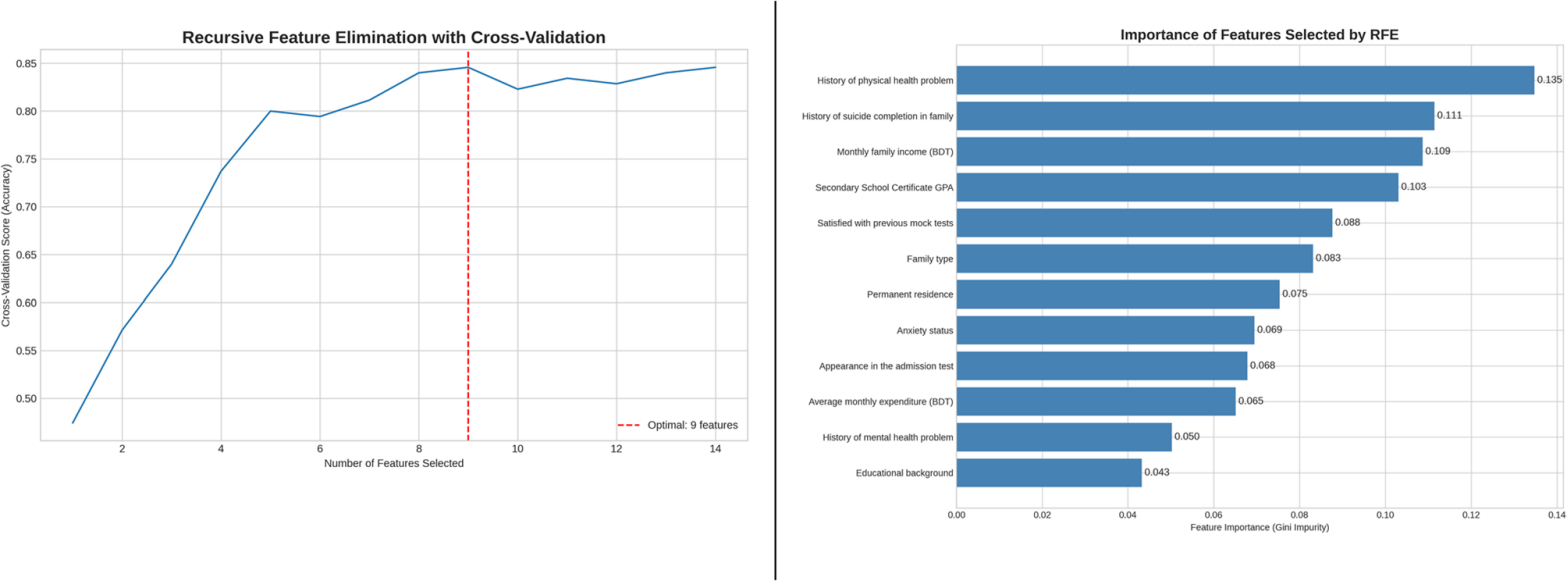
Feature Importance of the Optimal Subset Selected by RFE (Left). Model Performance versus Number of Features in Recursive Feature Elimination (Right)

To mitigate the risk of model overfitting and identify the most significant predictors from initial collection of 23 features was conducted a feature selection process. Which utilized Recursive Feature Elimination with 5-fold Cross-Validation (RFECV), employing a Random Forest classifier as the foundational estimator. This approach methodically eliminates the least significant features and assesses model performance at each stage, thus determining the ideal feature subset that enhances predicted accuracy. Figure 3 (Left) depicts the outcomes of the RFECV procedure. The cross-validation accuracy is graphed against the number of characteristics preserved. The performance reached its zenith with the utilization of 9 features, beyond which the accuracy stabilized, signifying that further features did not enhance predictive capability. Following this investigation identified these 9 best features for the construction of final prediction models. The Gini importance of the chosen feature subset is illustrated in Fig. 3 (Right). This ranking indicates that elements such as history of physical health problem (Gini value is 0.135) and history of suicide completion in family (Gini value is 0.111) were among the most significant predictors in the chosen feature set.

### LIME explanations for model interpretability

**Fig. 4:**
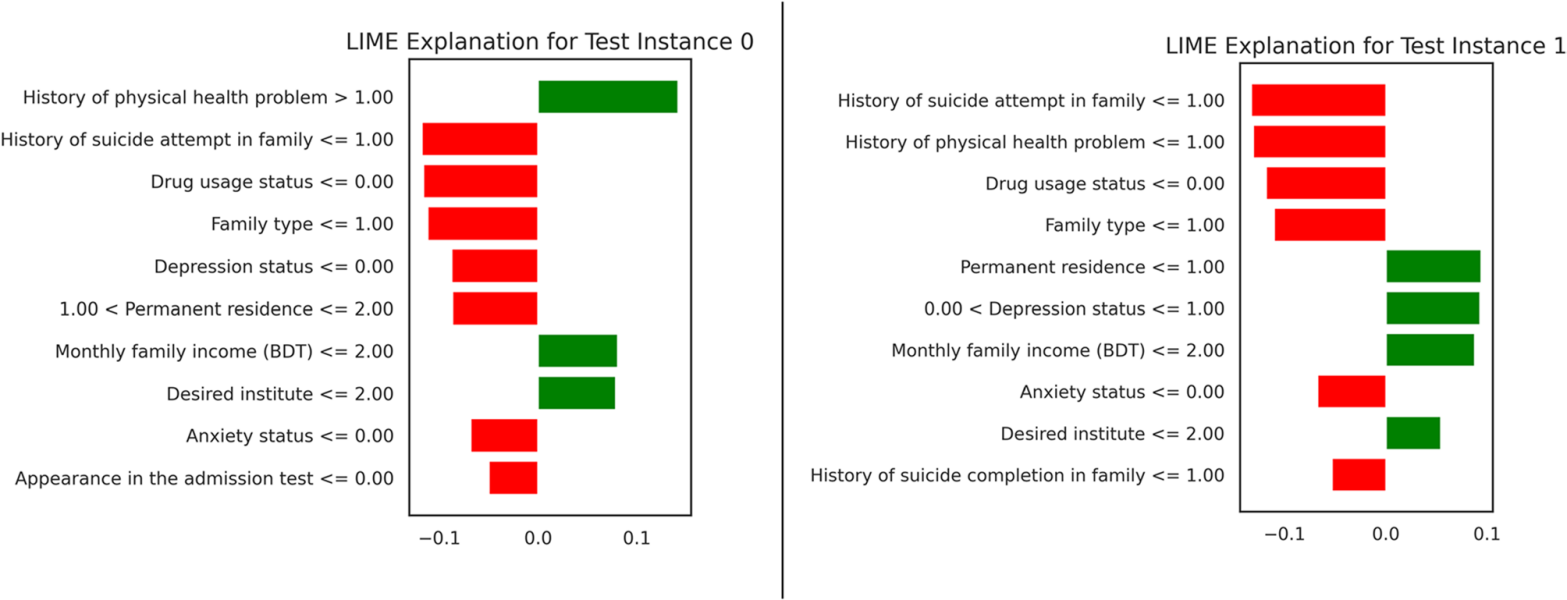
LIME Explanations for Test Instances 0 (No) and 1 (Yes)

In this part, presents Local Interpretable Model-Agnostic Explanations (LIME) explanations for two test situations to illustrate how specific features influence the model’s predictions made by the XGBoost model. Figure 4 illustrates the LIME-based visualizations for test cases 0 and 1. The graphs illustrate the most significant features for predictions, with green bars representing positive contributions (increased probability of ‘Yes’) and red bars denoting negative contributions (decreased probability of ‘Yes’). In Test Instance 0, a history of physical health problem and history of suicide attempt in family were critical factors contributing to the prediction of ‘Yes’ for suicidal thoughts. Conversely, factors such as family type and depression status exhibited negative contributions. These visualizations provide clarity regarding the model’s decision-making process, hence improving its interpretability and reliability.

### Learning curves for different Machine Learning model

**Fig. 5:**
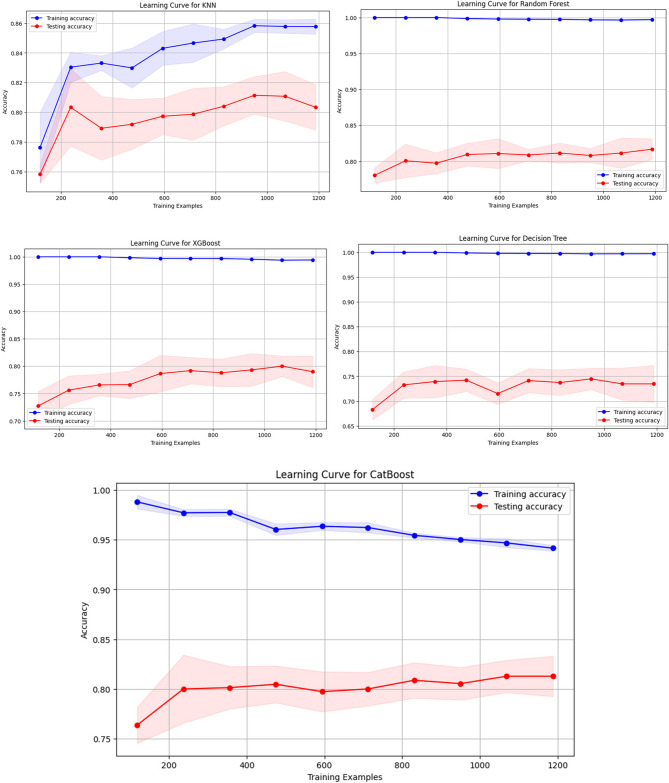
Learning Curves of Machine Learning Models

Figure 5 shows the learning curves for several machine learning models, such as KNN, RF, XGBoost, DT, and CatBoost. As the number of training instances rises, the curves show the models’ training and testing accuracy. All models generally maintain high training accuracy, suggesting that they match the training data well. Nonetheless, a discernible discrepancy between most models’ training and testing accuracy points to different levels of overfitting, in which the models perform noticeably better on the training data than the test data.

As the training set increases, the RF and DT models show very slight improvements in testing accuracy, indicating overfitting, despite having exceptionally high training accuracy and approaching perfect scores. With more data, KNN shows a steadier increase in testing accuracy, indicating a stronger generalization capability and comparatively lower variation. While the XGBoost and CatBoost models also exhibit great training accuracy, their testing accuracy is steady and might be further enhanced through regularization or hyperparameter tuning. Notably, hyperparameter tuning was applied to the RF and XGBoost models in an effort to improve generalization and decrease overfitting. In order to improve the balance between fitting the training data and generalizing to new data, these learning curves emphasize the necessity of meticulous tweaking and model selection.

### Evaluation of machine learning model performances

Table 3 presents the ML model’s prediction performance indicators for suicidal thoughts. After a thorough assessment that included measures such as accuracy, precision, F1 score, log-loss metrics AUC and 95% CI, each model was found to have differing levels of ability to predict outcomes associated to suicidal thoughts. Besides, all algorithms achieved respectable degrees of accuracy. KNN exhibited the highest accuracy score of 0.827, while CatBoost demonstrated 0.806. Regarding precision, KNN scored 0.813, while DT attained lowest 0.736. Additionally, in terms of F1-score KNN also provide the highest score 0.803. Except for DT, all algorithms showed logarithmic loss values of less than 3%, indicating extremely precise and secure model predictions. CatBoost achieved the lowest log loss of 0.412 and the highest AUC of 0.819, with a 95% confidence interval ranging from 0.779 to 0.860. However, The CatBoost model demonstrated the best performance in terms of log loss (0.421), AUC, and 95% confidence interval, indicating superior probability calibration and overall discrimination. While the KNN model also performed well in terms of accuracy, precision, and F1 score, its log loss (2.026) and AUC were slightly inferior to those of CatBoost, making CatBoost the most balanced and reliable model overall.

**Table 3:** Evaluation of machine learning model performances

Model	ACC	Precision	F1 score	Log loss	AUC	AUC 95% CI
KNN	0.827	0.813	0.803	2.026	0.742	0.693–0.791
RF	0.800	0.777	0.779	0.417	0.811	0.768–0.853
XGBoost	0.790	0.771	0.776	0.485	0.779	0.734–0.827
DT	0.722	0.736	0.729	9.594	0.624	0.576–0.675
CatBoost	0.806	0.785	0.783	0.412	0.819	0.779–0.860

5-fold cross-validation was used to evaluate each model on several data subsets in order to guarantee that the model assessments were solid and trustworthy. With this method, the risk of overfitting is reduced and a more accurate evaluation of the model’s performance across different data splits is obtained. Variations in model performance across various metrics and folds are shown in Table 4. With the highest average accuracy (0.786) and F1 score (0.786), CatBoost was the best-performing model, demonstrating robust and reliable performance. With an average accuracy of 0.785 and an F1 score of 0.786, RF also showed competitive performance, demonstrating its capacity to successfully strike a compromise between recall and precision. DT, on the other hand, showed the largest average log loss (10.273) and the lowest average accuracy (0.711), indicating that its forecasts were less confident and less dependable. The accuracy of KNN increased steadily during the folds, reaching a peak of 0.764 in Fold 4. However, KNN average Log Loss of 3.078 suggested that its forecasts were somewhat questionable. With an average accuracy of 0.774 and Log Loss of 0.627, which is marginally greater than CatBoost’s, XGBoost demonstrated steady performance, indicating less successful probability calibration. CatBoost’s average log loss is 0.501, with an AUC of 0.758 and a 95% confidence interval ranging from 0.684 to 0.825. In general, ensemble techniques such as CatBoost and RF outperformed individual models like DT in managing the complexity of the dataset.

**Table 4:** Performance of different models across 5-fold cross-validation

Model	Fold	ACC	Precision	F1 score	Log loss	AUC	AUC 95% CI
KNN	1	0.721	0.779	0.739	3.502	0.736	0.661–0.798
	2	0.71	0.768	0.73	3.766	0.722	0.647–0.796
	3	0.751	0.795	0.766	3.827	0.739	0.661–0.811
	4	0.764	0.801	0.777	1.931	0.799	0.730–0.857
	5	0.721	0.765	0.736	2.362	0.722	0.650–0.793
KNN Average	--	0.733	0.782	0.750	3.078	0.744	0.670–0.811
RF	1	0.788	0.787	0.787	0.681	0.791	0.722–0.848
	2	0.761	0.773	0.766	0.504	0.758	0.691–0.818
	3	0.785	0.794	0.789	0.477	0.808	0.742–0.865
	4	0.818	0.809	0.813	0.416	0.830	0.767–0.882
	5	0.774	0.778	0.776	0.478	0.772	0.707–0.835
RF Average	--	0.785	0.788	0.786	0.511	0.792	0.726–0.850
XGBoost	1	0.781	0.782	0.782	0.614	0.743	0.672–0.811
	2	0.754	0.784	0.765	0.663	0.731	0.659–0.798
	3	0.754	0.769	0.761	0.66	0.737	0.664–0.811
	4	0.805	0.801	0.802	0.528	0.775	0.702–0.835
	5	0.774	0.771	0.773	0.668	0.699	0.62–0.772
XGBoost Average	--	0.774	0.781	0.777	0.627	0.737	0.663–0.805
DT	1	0.684	0.723	0.7	11.41	0.584	0.518–0.651
	2	0.694	0.738	0.71	10.807	0.616	0.548–0.686
	3	0.697	0.736	0.712	10.805	0.631	0.564–0.697
	4	0.747	0.754	0.751	8.984	0.633	0.572–0.698
	5	0.731	0.766	0.744	9.359	0.652	0.586–0.716
DT Average	--	0.711	0.743	0.723	10.273	0.623	0.558–0.690
CatBoost	1	0.795	0.798	0.796	0.505	0.715	0.692–0.823
	2	0.724	0.751	0.735	0.563	0.715	0.64–0.784
	3	0.795	0.808	0.8	0.498	0.770	0.698–0.842
	4	0.825	0.816	0.819	0.429	0.797	0.724–0.859
	5	0.791	0.791	0.791	0.509	0.744	0.668–0.817
CatBoost Average	--	0.786	0.793	0.786	0.501	0.758	0.684–0.825

The performance of 5 machine learning models was compared in this study using their AUC (Area Under the Curve) ratings, which were acquired in Table 3. In order to ascertain the statistical significance of the model differences, we calculated the AUC difference and associated 95% CI for each set of models using a bootstrapping technique. The AUC performance of different model pairs varies significantly, according to the data. For instance, DT demonstrated a notable gain in performance when compared to other models, but KNN displayed a lower AUC than RF, XGBoost, and CatBoost. Although the AUC values of XGBoost and CatBoost were rather comparable, there was a noticeable performance difference when compared to DT. The estimations’ relative precision was demonstrated by the narrow confidence intervals for the AUC differences in each comparison. With notable AUC differences observed in certain model comparisons, the results indicate that Random Forest and CatBoost outperform the other models. Table 5 displays the bootstrapped AUC differences for each pairwise model comparison.

**Table 5:** AUC differences and 95% confidence intervals for pairwise model comparisons

Model 1	Model 2	AUC Difference	95% CI Lower	95% CI Upper
KNN	RF	− 0.069	− 0.069	− 0.069
	XGBoost	− 0.037	−0.037	− 0.037
	DT	0.118	0.118	0.118
	CatBoost	− 0.077	−0.077	− 0.077
RF	XGBoost	0.032	0.032	0.032
	DT	0.187	0.187	0.187
	CatBoost	− 0.008	−0.008	− 0.008
XGBoost	DT	0.155	0.155	0.155
	CatBoost	− 0.040	−0.040	− 0.040
DT	CatBoost	− 0.195	− 0.195	− 0.195

**Fig. 6:**
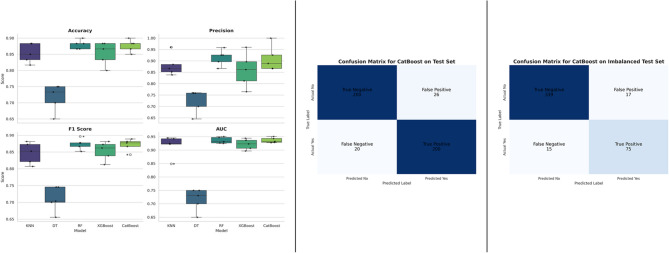
Comparative Model Performance and Final Test Set Evaluation

To rigorously evaluate the candidate models, which initially conducted a 5-fold cross-validation. Figure 6 (Left) depicts the distribution of performance measures for each model. The findings indicate that although Random Forest and XGBoost exhibited robust performance, the CatBoost model attained the highest median performance and stability, especially regarding Area Under the Curve (AUC), with a mean AUC of 0.966 (SD = 0.019). According to these findings, CatBoost was identified as the superior model. Subsequently, we trained it on the complete training dataset and assessed its ultimate performance on the unseen, held-out test set. The CatBoost model attained an mean Accuracy of 87.7%, a Precision of 91.0%, and an F1-Score of 87.10% on this imbalanced test dataset. The resultant confusion matrix is illustrated in Fig. 6 (Middle). This comprehensive assessment, demonstrating 200 True Positives and merely 20 False Negatives, validates the model’s robust capacity to accurately recognize the minority class and generalize proficiently to novel data. The completed CatBoost classifier was assessed on the reserved, imbalanced test set to validate its real-world performance. Figure 6 (Right) displays the resultant confusion matrix. This is evidenced by the 75 accurately recognized positive cases (True Positives) compared to merely 15 overlooked ones (False Negatives), hence affirming the model’s efficacy and generalizability on novel data.

**Fig. 7:**
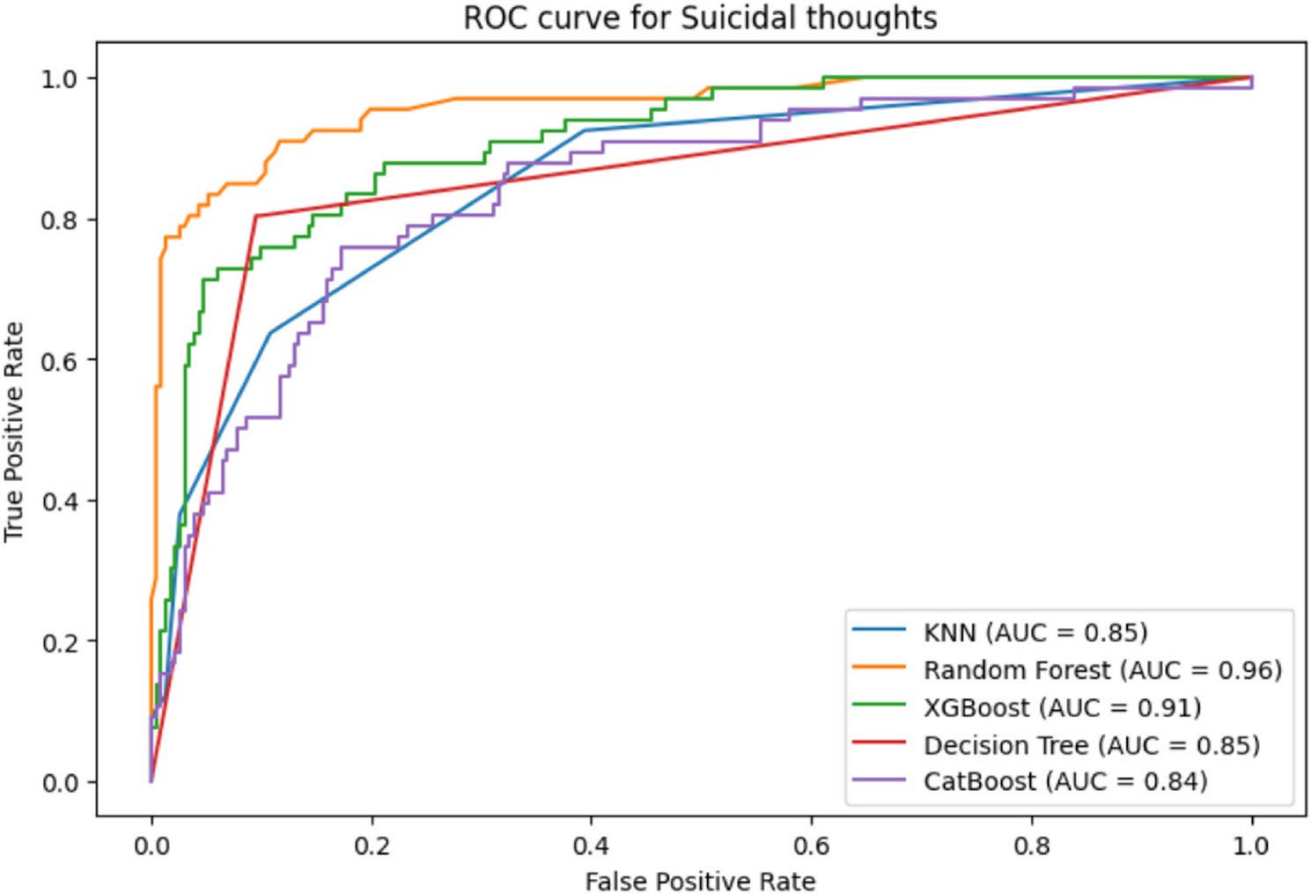
ROC-AUC curve of suicidal thoughts

Figure 7 showed the algorithms’ Receiver Operating Characteristic Curve (ROC-AUC), a critical assessment parameter in Machine Learning for binary classification models. A model’s capacity to distinguish between positive and negative classifications is evaluated using ROC-AUC. In the RF model shows remarkable accuracy in differentiating between positive and negative classifications, with an AUC score of 0.96. XGBoost exhibits strong discriminatory power, as evidenced by its maximum AUC score of 0.91. Unfortunately, the CatBoost model yields an AUC score of 0.84 which is the lowest scores. Overall, RF show the strongest discriminatory power of all models based on their AUC values, effectively and competently differentiating between positive and negative classifications.

## Discussion

This study represents a comprehensive endeavor to not only gauge the prevalence and risk factors of suicidal thoughts among university entrance test-taking students but also to explore novel avenues in predictive modeling through the integration of GIS mapping and Machine Learning techniques. Guided by the Stress-Diathesis Model, which conceptualizes suicidality as the interaction of external stressors and individual vulnerabilities, this study merges traditional statistical methods with machine learning and GIS approaches to offer a holistic understanding of the factors contributing to suicidality. This integrated framework not only strengthens the theoretical grounding of the findings but also paves the way for more targeted and effective intervention strategies and support systems.

This study marks a significant departure from prior studies by delving into the landscape of suicidal behaviors among university entrance test-taking students in the post-pandemic era, an area that remains largely unexplored. Previous studies conducted before and during the pandemic within the test-taking students revealed varying prevalence rates of suicidal ideation, planning, and attempts. Specifically, pre-pandemic rates stood at 17.7% for suicidal thoughts, 8.0% for plans, and 2.5% for attempts [11], whereas during the pandemic, these rates declined slightly to 14.4%, 7.4%, and 7.2%, respectively [13]. But this study unveils a noteworthy shift in the post-pandemic landscape, with 20.5% of the participants reporting suicidal thoughts within the past year. Contrary to expectations, while the prevalence of suicidal thoughts decreased after the pandemic’s onset, attempts to complete suicide increased. The overall prevalence of suicidality observed in this study surpasses that of previous investigations, which highlights the urgency and significance of this study findings in the context of post-pandemic mental health.

Gender emerges as a significant factor influencing suicidality, with females exhibiting a 1.675 times higher risk compared to males. This aligns with findings from pre-pandemic study [11], although a non-significant relationship was observed during the pandemic [13]. Likewise, urban residence emerges as a consistent risk factor across all three studies. Interestingly, students from joint families displayed higher rates of suicidal behavior, contrasting with the lower risk observed among middle-class families in this study. In terms of admission-related variables, participants with poor academic performance and those not coached by professional centers also displayed elevated risks. Aspiring for admission to agricultural subjects was associated with an elevated risk of suicidal thoughts. Moreover, substance use, mental health issues, and family mental health and suicide history increased odds of suicidal thoughts. Our findings illustrated Machine Learning highlighting depression status as the most impactful predictor, while family history of suicide had the least effect. Several non-linear relationships where factors such as sociodemographic factor, admission related information and mental health problems interact in complex ways, significantly influencing the risk of suicidal thoughts. SHAP analysis revealed that the relationship between the studied factors and suicidal thoughts is non-linear, with risk increasing exponentially as stress accumulates beyond a certain threshold. Additionally, interaction effects visualized through SHAP showed that students with a combination of high academic pressure and insufficient sleep were disproportionately at risk, suggesting a multiplicative effect of these factors on suicidal ideation. This study’s findings echoed the previous study conducted within Korean middle school students from 2011 to 2019 was used for predictors of suicide ideations and found that mental health was the most important factor suing the naive Bayes classifier [24]. The application of Machine Learning enhances understanding and prediction of suicidal behaviors, highlighting the need for further research and targeted interventions in this area, particularly focusing on the most impactful factors. Addressing these risk factors and fostering a supportive environment are crucial for reducing suicidality risk and enhancing overall well-being. Integrated healthcare approaches addressing both physical and mental health needs are essential to mitigate suicidality risks among vulnerable populations.

Consistent with findings from the previous studies [11, 13], this investigation reveals a 1.75 times higher risk of experiencing suicidal thoughts among students undertaking the university entrance test for the second time. Specifically, a notable prevalence of suicidal behavior was observed among repeat test-takers, with 27.1% reporting suicidal thoughts compared to 17.5% among fresher test-takers. This trend mirrors observations from pre-pandemic and pandemic-era studies [11, 13], where repeat test-takers consistently exhibited higher rates of suicidal behaviors compared to their first-time counterparts. While some argue that repeat test-takers may benefit from additional preparation time, potentially leading to increased confidence and psychological stability, a number of studies within this population paint a contrasting picture. Despite the debate surrounding their potential success, repeat test-taking students reported a higher prevalence of psychological problems, including depression, anxiety, and other psychological issues [11]. These findings emphasize the urgent need for targeted interventions to support the mental well-being of students, particularly those facing academic challenges and repeated test attempts.

The study’s findings shed light on the multifaceted factors influencing suicidal thoughts among university entrance test-taking students, particularly in the realm of health and behavioral variables. The prevalence of suicidality among participants with a history of physical health problems underscores the intricate relationship between physical and mental well-being. Chronic physical health conditions may contribute to psychological distress, exacerbating existing mental health challenges and elevating the risk of suicidal behavior [39]. Besides, participants engaging in substance use, including smoking cigarettes, using drugs, and consuming alcohol, exhibited significantly higher rates of suicidal thoughts. This highlights the significant role of substance abuse as a contributing factor to suicidality within this demographic. Previous research has also linked early initiation of substance use to an increased susceptibility to suicidality [40], while various unhealthy lifestyle behaviors have been identified as predictive factors for suicide attempts [41, 42].

Individuals with a history of mental health problems demonstrated a significantly elevated prevalence of suicidality compared to those without such a history. This aligns with existing literature emphasizing the association between mental health disorders and suicidal behaviors, highlighting the importance of early detection and intervention in addressing mental health concerns among university students [17]. Evidently retrospective studies from Bangladesh report students’ suicide reasons to be mental health and associated issues closely linked with emotional issues, conflict with family, relationship, and sexual problems, etc [43]. Previously, up to four times higher risk of suicidal thought was found in those who were depressed or anxious [11], and a similar risk of suicidal ideation was reported in this study. Depression status emerged as the most impactful predictor of suicidal thoughts according to the Machine Learning model, although a family history of suicide had the least effect. Moreover, compelling associations were found between familial factors and suicidal behavior. Participants with a family history of mental health problems, suicide attempts, or suicide exhibited significantly higher rates of suicidal behavior. These findings highlight the potential influence of familial dynamics and genetic predispositions on an individual’s susceptibility to suicidal thoughts and actions [31]. Understanding the role of family history can inform targeted interventions aimed at addressing familial risk factors and providing support to vulnerable individuals within familial contexts.

This study reveals significant variations in suicidal ideation across various districts of the country as per the findings found in GIS analysis. Particularly high prevalence rates of suicidality were observed in specific districts, notably those within the Sylhet division, such as Sunamganj, Sylhet, and Maulovibazar. Elevated rates were also identified in other districts like Naogaon, Natore, Shariatpur, Pirojpur, Cox’s Bazar, and those within the Chittagong Hill Tracts, including Khagrachori and Rangamati. Although the distribution of suicidality rates in the Chittagong Hill Tract resembled the previous study, differences were noted in other regions. Previously, areas lacking modern facilities, including education-related facilities and transportation, were more likely to report suicidal thoughts, but the current findings present a nuanced perspective [13]. Several factors may contribute to these variations, highlighting the need for a more in-depth spatial analysis that incorporates environmental and socio-economic factors into the GIS model. Future research could strengthen this analysis by incorporating additional data layers, such as healthcare accessibility, economic conditions, and social support systems, to provide deeper insights into the contextual factors influencing suicidality. Using GIS data in this manner can be a valuable tool for developing targeted suicide prevention programs. However, achieving a comprehensive understanding will require further exploration of the complex interactions between spatial patterns and socio-economic determinants.

In terms of Machine Learning, the CatBoost model demonstrated strong performance, achieving high accuracy rates of 0.806, AUC value of 0.819 and 95% CI range of 0.779–0.860 and low log loss values of 0.412. Besides, CatBoost exhibited precision values of 0.783, while KNN reached the highest precision value of 0.813. KNN also boasted the highest F1 score at 0.803, with CatBoost showing respectable ratings at 0.783. Except for Decision Trees, which displayed a loss value of 9.594, all other algorithms maintained logarithmic loss rates of less than 3. Notably, CatBoost demonstrated the largest log loss at 0.412, indicating its predictive accuracy. Concerning AUC values, RF exhibited the strongest discriminatory power with scores of 0.96, while XGBoost also performed well with a maximum AUC score of 0.91. Additionally, the KNN model demonstrated strong generalization and consistent convergence of training and validation accuracy in learning curves across various machine learning models. Similarly, the CatBoost model exhibited a continuous increase in validation accuracy, albeit with an initial decline in training accuracy. In the 5-fold cross-validation, CatBoost achieved an average accuracy of 0.786, precision of 0.793, and F1-score of 0.786 with the lowest log loss was 0.501, AUC of 0.758 and a 95% confidence interval ranging from 0.684 to 0.825. Overall, the performance of the CatBoost model surpassed that of other models, underscoring the importance of efficient interventions to enhance the health and well-being of students.

Machine Learning techniques have revolutionized the analysis of suicidal behavior by enhancing prediction accuracy and classification in numerous studies. For example, a systematic review of 1032 studies employing machine learning techniques reported accuracies of 70% or higher in assessing suicide risk or in predicting suicidal attempts [21]. These findings resonate with previous original studies, particularly a study by Weller et al. [30], which highlighted the efficacy of ML in predicting suicidal thoughts and behavior among adolescents, achieving an accuracy of 91% by the tree-based model, LightGBM. Similar to our findings, their study identified critical predictors such as familial dynamics and peer acceptance. In a study among Chinese adolescents of first or second-year middle school and high-school, the Random Forest model exhibited superior accuracy in predicting suicidal ideation compared to other models, with a mean accuracy of 87.3% and an AUC of 92.4% [26]. Similarly, among middle school students in Korea utilized a machine-learning approach to predict suicide ideation, a decision tree, logistic regression, and a naive Bayes classifier were applied, and found that Logistic Regression achieved the highest validation ROC score of 0.82 [24]. In another comparison, the findings align with those of Macalli et al. [29], who developed a risk algorithm for college students, reporting an AUC of 0.80 and identifying key predictors, including prior suicidal thoughts and anxiety. In a longitudinal study of individuals aged 14 to 34 in France, Miché et al. (2020) reported mean AUCs for four predictive models: logistic regression (0.828), lasso (0.826), ridge (0.829), and random forest (0.824) [25]. Our study’s identification of anxiety and depression as significant predictors supports their findings, illustrating the consistency in predictive factors across different demographics. Furthermore, the results reflect Kirlic et al. [44], who emphasized the role of social isolation and positive affect as critical components in understanding suicidal thoughts among college students, suggesting that these factors are universally relevant in various ML contexts. These studies collectively emphasize the effectiveness of ML techniques in improving our understanding and prediction of suicidal behavior, highlighting their potential in guiding targeted interventions and support services for at-risk individuals.

### Implications of machine learning for suicide prevention

The findings advance the literature on Machine Learning’s efficacy in mental health research by identifying key predictive factors related to suicidal ideation in a Bangladeshi context. The success of the CatBoost model indicates that integrating advanced ML algorithms can refine theoretical frameworks for assessing mental health risks, enhancing our understanding of predictors of suicidal behavior in adolescents and young adults. Practically, this study highlights the need for targeted interventions based on these risk factors. Integrating ML models into routine mental health screenings can improve early identification of at-risk individuals, enabling mental health professionals to develop personalized intervention strategies that foster resilience and enhance overall well-being.

In addition to their predictive utility, these findings align strongly with the Stress-Diathesis Model, a well-established theoretical framework that conceptualizes suicidal behavior as arising from the interaction between environmental stressors and individual vulnerabilities. In this study, ML algorithms revealed that external stressors—such as repeated test-taking, poor academic performance, and familial pressure—combined with internal vulnerabilities, including depression, anxiety, and substance use, were key contributors to suicidal ideation. This convergence between data-driven insights and theoretical understanding strengthens the model’s applicability to the Bangladeshi context. Moreover, it illustrates how ML can be used to operationalize abstract psychological constructs, translating them into measurable variables for prediction and intervention. By capturing this stress–vulnerability interaction at scale, ML-enhanced tools grounded in psychological theory can substantially improve the precision and practical relevance of suicide risk assessment.

To maximize the impact of the machine learning models developed in this study, practical deployment strategies are essential; schools and universities could incorporate these models into their mental health initiatives, using them in screening processes to identify students at risk of suicidal ideation. Regular training for mental health professionals on interpreting and utilizing ML outputs could further enhance the effectiveness of these interventions, while fostering awareness and support systems for students could create a more inclusive environment where individuals feel empowered to seek help. By addressing the predictors identified in this study, educational institutions can play a pivotal role in suicide prevention efforts, ultimately improving the mental health landscape for students.

### Limitations and ethical considerations of the study

This study employs a combination of GIS mapping and Machine Learning techniques to uncover the spatial distribution and predictive factors of suicidal thoughts among university entrance test-taking students in Bangladesh, offering a comprehensive approach to understanding this intricate phenomenon. However, this focus on a specific student population from a single university limits the generalizability of the findings to other populations or regions. Important contextual factors such as geographical location, living environment, and teaching level were not included in the analysis, which may affect the model’s broader applicability. While the ML models developed provide valuable insights, their transferability to different cultural or educational contexts will require further validation. The integration of ML algorithms enhances the accuracy of predictions, enabling the identification of subtle patterns and risk factors associated with suicidality. Nonetheless, this enhancement introduces challenges such as model interpretation and potential overfitting. Besides, the study’s reliance on self-reported data may be subject to recall bias, and the cross-sectional design limits the ability to establish causality. Moreover, suicidal ideation was assessed over a one-year period prior to the university entrance exam. We acknowledge that this timeframe is relatively broad, and students’ mental health experiences may vary significantly at different stages of the preparation process. This limitation may reduce the temporal precision of our findings. Future research should consider dividing this period into shorter intervals to capture fluctuations in suicidal ideation and better identify stage-specific influencing factors.

Ethical considerations are paramount when applying ML to mental health research, particularly for vulnerable populations. Researchers must prioritize informed consent, data privacy, and the potential stigmatization of individuals identified as at risk. It is essential to navigate concerns related to permission, data handling, and the risk of misclassification to avoid harm. Fairness and transparency are critical, as ML models may inadvertently reflect biases present in the data [19]. Collaborative efforts involving mental health professionals, ethicists, and data scientists can guide the responsible use of technology in mental health research. Rather than functioning autonomously, ML models should serve as decision-support tools to assist mental health professionals in assessing risk and providing timely interventions, thereby respecting the dignity and autonomy of participants while addressing sensitive topics like suicidal ideation [45]. Despite these limitations, the integration of Machine Learning with GIS mapping represents a pioneering advancement in suicide research, paving the way for more precise risk assessment and targeted intervention strategies.

## Conclusions

In conclusion, this study offers a comprehensive understanding of suicidal thoughts among university entrance test-taking students in Bangladesh, revealing a concerning increase in post-pandemic suicidal ideation. By elucidating the associations between health, behavioral, and familial factors, the findings provide valuable insights for the development of comprehensive prevention and intervention strategies tailored to address the unique needs of this population. The successful application of Machine Learning techniques, notably the CatBoost model, highlights the value of advanced analytical tools in identifying vulnerable individuals with precision. To effectively address the complex drivers of suicidal behaviors, concerted efforts are needed to raise mental health awareness, curb substance abuse, and bolster familial support systems. As we move forward, prioritizing proactive mental health support and tailored interventions remains imperative to cultivate a nurturing and resilient environment for university students, both locally and globally.

## Data Availability

The data that support the findings of this study are available from the corresponding author upon reasonable request.
